# Novel Acid-Assisted Polymerization Technique for the Synthesis of Polyaniline Films at Room Temperature on Glassy Carbon for Supercapacitor Applications

**DOI:** 10.1021/acsomega.5c02109

**Published:** 2025-05-13

**Authors:** Stephen Boahene, Štěpán Potocký, Kateřina Aubrechtová Dragounová, Ondrej Szabó, Elena Tomšík, Alexander Kromka

**Affiliations:** † 679861 Czech Technical University in Prague, Faculty of Electrical Engineering, Technická 1902/2, Prague 6 166 27, Czech Republic; ‡ Institute of Physics of the Czech Academy of Sciences, Cukrovarnická 10, Prague 6 162 00, Czech Republic; § Institute of Macromolecular Chemistry AS CR, Heyrovsky nam. 2, Prague 6 162 00, Czech Republic

## Abstract

This study presents polyaniline (PANI) synthesis and characterization by using a novel acid-assisted polymerization technique. Two PANI suspensions with different ammonium peroxydisulfate (APS) concentrations were synthesized at room temperature; i.e., the ratio of aniline to APS was 10:1 for PANI1 and 5:1 for PANI2. SEM measurements revealed distinct structures: a porous nanofibrillar structure for PANI1 and a densely packed structure for PANI2. The electrochemical performance of the fabricated PANI/glassy carbon (GC) electrodes was evaluated using a three-electrode cell configuration at scan rates of 10 and 30 mV/s. The PANI1/GC heterostructure exhibited a specific capacitance of 160 F/g, while this value increased to 407 F/g for the PANI2/GC. This research contributes not only to the understanding of PANI synthesis at room temperature but also to its potential applications in electrochemical energy storage devices.

## Graphical abstract

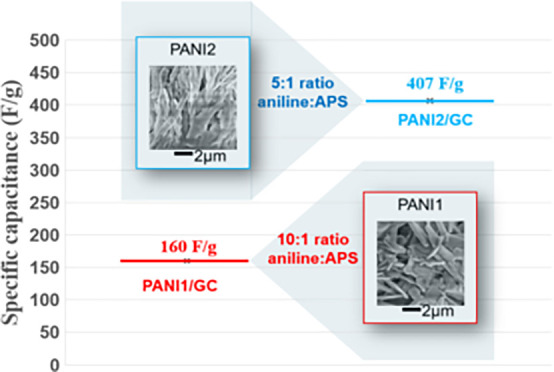

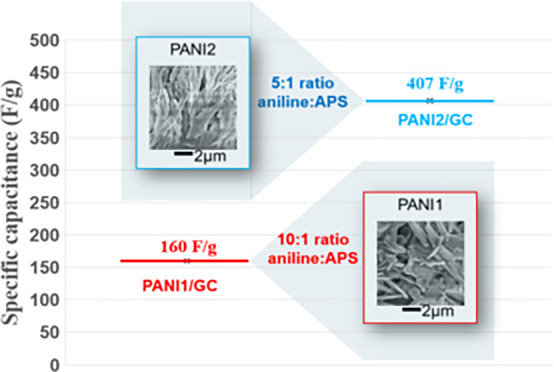

## Introduction

The increasing demand for readily available energy necessitates the development of efficient and sustainable energy storage technologies. Due to their high-power density with fast charging/discharging capabilities, exceptional cycle life, and environmental benefits, electrode-based energy storage devices, particularly supercapacitors, have attracted a lot of attention.^1^ A promising research area involves developing and fabricating new solar-driven (or photoassisted) composite electrodes for high-performance asymmetric supercapacitor applications.^2,3^ These innovative materials integrate solar energy harvesting capabilities with electrochemical storage functions to enable self-charging operations and superior energy storage capabilities compared to other electrode materials.^2,3^

Supercapacitors are predominantly classified into three primary groups based on their charge storage mechanisms, including electric double-layer capacitors (EDLCs), pseudocapacitors, and hybrid capacitors.^4,5^ EDLCs store energy through electrostatic adsorption and desorption of ions at the interface between a high-surface-area electrode material and an electrolyte, contributing to their long cycle life via a non-Faradic process that facilitates rapid charge and discharge cycles.^5,6^

The electrochemical performance of supercapacitors in a specific application is predicated on their constituent electrode materials and charge storage mechanisms. Furthermore, as there is a need for supercapacitors to require high-power characteristics, the electrode material needs to have both a large surface area and good electronic conductivity.^7^

Carbonaceous nanomaterials, including carbon nanotubes, activated carbon, and graphene, characteristically exhibit EDLC behavior, offering advantages such as long-life cycle and high electrical conductivity, albeit with comparatively modest charge storage capacities.^8^ Pseudocapacitors, on the other hand, utilize Faradic redox reactions at the electrode surface to store energy through the transfer of electrons between the electrode material and the electrolyte, offering higher specific capacitance values compared to those of EDLCs. Common electrode materials, such as metal oxides (e.g., MnO_2_, V_2_O_5_, RuO_2_) and conducting polymers (e.g., polyacetylene, polypyrrole, polyaniline), demonstrate reversible and rapid Faradic reactions that significantly enhance charge storage capacities.^5,8,9^

However, the practical implementation of purely pseudocapacitive materials is often constrained by limitations in long-term stability and rate performance. Hybrid supercapacitors represent the third category, combining EDLCs and pseudocapacitive characteristics to optimize both energy density and power density.^8,10^ This approach aims to overcome the inherent trade-offs associated with single-component electrode fabrication of composite materials that synergistically integrate the desirable attributes of both EDLC and pseudocapacitive materials. Consequently, comprehensive global research efforts are focused on exploring different material combinations for flexible energy storage device applications.^8^

Conjugated organic molecules and their polymers, also known as functional π-conjugated systems, have become a key group of materials. Their unique optoelectronic properties are characterized by tunable energy levels and efficient transport of charges. These materials offer additional advantages including lightweight composition, easy processing in solutions, and adaptable material design. As mentioned, their cost-effective production and their appeal in scientific and technological fields are growing rapidly.^11,12^ This demand for conjugated (semiconducting) polymers with specific morphologies and properties tailored for particular device applications necessitates the development of simple and reliable synthesis methods.

From a large family of such conjugated polymers, polyaniline (PANI) has garnered considerable scientific interest since conducting polymers were discovered. It has been widely recognized for its diverse range of potential applications (e.g., gas sensors, catalysis, composite fabrication, adsorption, energy storage devices, and many others) and its notable advantages, encompassing facile synthesis, cost-effectiveness, robust environmental stability, and exceptional performance characteristics.^13^

PANI demonstrates enhanced charge storage capabilities when synthesized as nanofibers, nanotubes, or other porous structures with high surface area, potentially increasing specific capacitance.^11^ As a redox-active material, PANI can store energy through Faradic processes. However, PAN’s inherent limitations include mechanical integrity and electrical conductivity. A significant challenge in PANI applications is the structural disorder that blocks the diffusion of the electrolyte into the polymer matrix.^11^ To mitigate these challenges and improve device performance, researchers have explored composite materials incorporating carbon materials, metal oxides, and alternative polymers. Such combinations can greatly enhance the rate of ionic transport as well as the mechanical stability of the electrode.^11,14–17^

Current PANI synthesis techniques include chemical oxidation polymerization,^18^ electrochemical polymerization,^19^ metal-catalyzed coupling,^20^ solid-state polymerization,^21^ Lewis acid-assisted polymerization,^22^ acid-assisted polymerization,^1,23^ photoinduced polymerization, and high-temperature oxidant-free acid polymerization.^24^ Its synthesis depends on several factors such as oxidant, solvent, electrode material, pH, temperature, and presence of chemical additives (like oligoaniline and π-bonding compounds), as well as the composition of the electrolyte and dopant anions.^25^ These factors modulate the PANI molecular structures and properties, necessitating careful optimization to obtain specific physicochemical properties that enhance the performance in various applications. Chemical oxidative polymerization is the primary approach for producing PANI with a controlled chemical structure, size of the chains, and morphology, showing a promising strategy for industrial synthesis. PANI can be synthesized by oxidative polymerization using ammonium persulfate (APS) as an oxidant in strongly acidic solutions. Nevertheless, it is well-known that PANI cannot be obtained in a weakly acidic medium, as the oxidation of aniline at a higher pH leads to oligomer formation.^26,27^ However, recent studies have introduced a novel synthesis approach for fabricating sensing layers utilizing PANI films and chelating agents.^23^ Aniline was polymerized using the acid-assisted polymerization method, where an acidic solution (concentrated formic acid) was used as a medium alongside APS as the initiator of the reaction. The novel synthetic approach, the acid-assisted polymerization technique, leverages formic acid to facilitate ion-electron transfer reactions between monomer units. This method enables the formation of stable polymer suspensions, including poly­(3,4-ethylenedioxythiophene), PEDOT, polypyrrole (PPy), and polyaniline.^28–30^ This synthesis surpassed other traditional PANI synthesis methods by achieving full conversion of the monomer in a 24 h period at ambient temperature, producing a stable solution; however, at a slow pace.

The pursuit of the best electrode materials for flexible supercapacitors focuses on achieving exceptional electrochemical performance characterized by high capacitance, robust stability, remarkable flexibility, and compact architecture. These attributes demand electroactive materials with a high specific surface area, excellent electrical conductivity, and a porous framework, facilitating facile electrolyte ion transport. In this context, Ahirrao et al. recently reported the development of nanostructured porous polyaniline-coated carbon cloth (CC) as electrodes for a flexible supercapacitor device.^8^ The carbon-based materials, renowned for their robust mechanical integrity, efficiency on transport pathways, and high electrical conductivity, are extensively investigated as a promising electrode platform.^31^ Furthermore, recent studies showed an application of coal-based graphitic carbons where coal was converted to carbon nanomaterials in the application of energy storage devices.^32^ The scientific exploration of conjugated polymers and their carbon-based composites continues to reveal promising avenues for advanced energy storage devices, where the porosity of carbon-based materials provides a large specific surface area and the resulting porous carbon electrode exhibits a high specific surface area, yielding an area capacitance with enormous energy and power densities.^33^

The current study aims to elucidate the time frame required for a complete synthesis of PANI and, subsequently, perform a comprehensive characterization of the resultant PANI solution. Glassy carbon’s (GC) physical and chemical characteristics have made it an intriguing and often-used electrode material. GC is an inert electrode used due to its relatively low oxidation rate, good chemical inertness, very small pore diameters, and reduced gas and liquid permeability,^34^ making it a favorable material for electrocatalytic applications. The physicochemical characteristics of GC electrodes are significantly influenced by the initial polymer and the carbonization temperature.^35^ As they are shown to be one of the most intriguing and modified electrodes in electrochemical measurements with improved electrocatalytic performance, GC can be activated by several procedures, including vacuum heating, treatment with laser, mechanical polishing, carbon arc, and ultrasonication.^36^

In this study, we focus primarily on the PANI synthesis by the method with two different APS concentrations (PANI1 and PANI2), the characterization of PANI morphologies, and the application of cyclic voltammetry (CV) and electrochemical impedance spectroscopy (EIS) methods to evaluate the electroactive performance of PANI for supercapacitor applications.

## Results and Discussion

In this study, stable PANI suspensions were successfully synthesized using the acid-assisted polymerization method.^23^ Two PANI suspensions with different initiator loadings of APS were prepared, as illustrated in Figure 1. The stoichiometric feeding ratio of aniline to APS has been reported to influence the synthesis of different PANI forms.^26^

**Figure 1:**
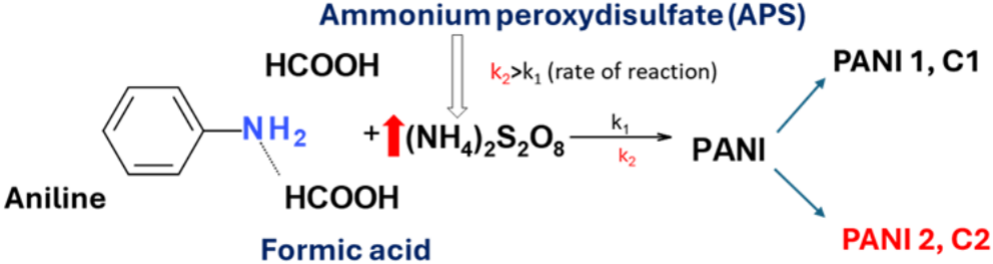
Schematic diagram of the preparation process of polyaniline (PANI1 and PANI2). The reaction kinetics are characterized by reaction constants *k*_1_ and *k*_2_.

### Raman Spectroscopy of Glassy Carbon (GC)

The intention of measuring the Raman spectra of GC was to prove that the substrate does not affect the Raman spectra of PANI. Using a 785 nm excitation wavelength, we observed two prominent peaks in the Raman spectrum of GC (Figure 2). The peaks located at approximately 1310 cm^–1^ (D band) and 1610 cm^–1^ (G band) are characteristic features of graphitic carbon structures.^37^ The D band, associated with disorder-induced Raman scattering, is absent in perfect single-crystal graphite.^38^ Recent studies suggest that Raman scattering by double resonant is a likely mechanism for the origination of D bands.^39^ The G band, coming from an in-plane stretching vibration of sp^2^-bonded carbon atoms, indicates the presence of graphitic-like structures.^40^ The relative intensity ratio of the D and G bands (*I*_D_/*I*_G_) and the line width of the D band are key indicators of the sample’s graphitic quality and defect density. In addition to D and G bands, the second-order Raman peaks near 2600 and 2900 cm^–1^ are also well recognizable and correspond to the 2D peak and G + D peak combination.^40,41^

**Figure 2:**
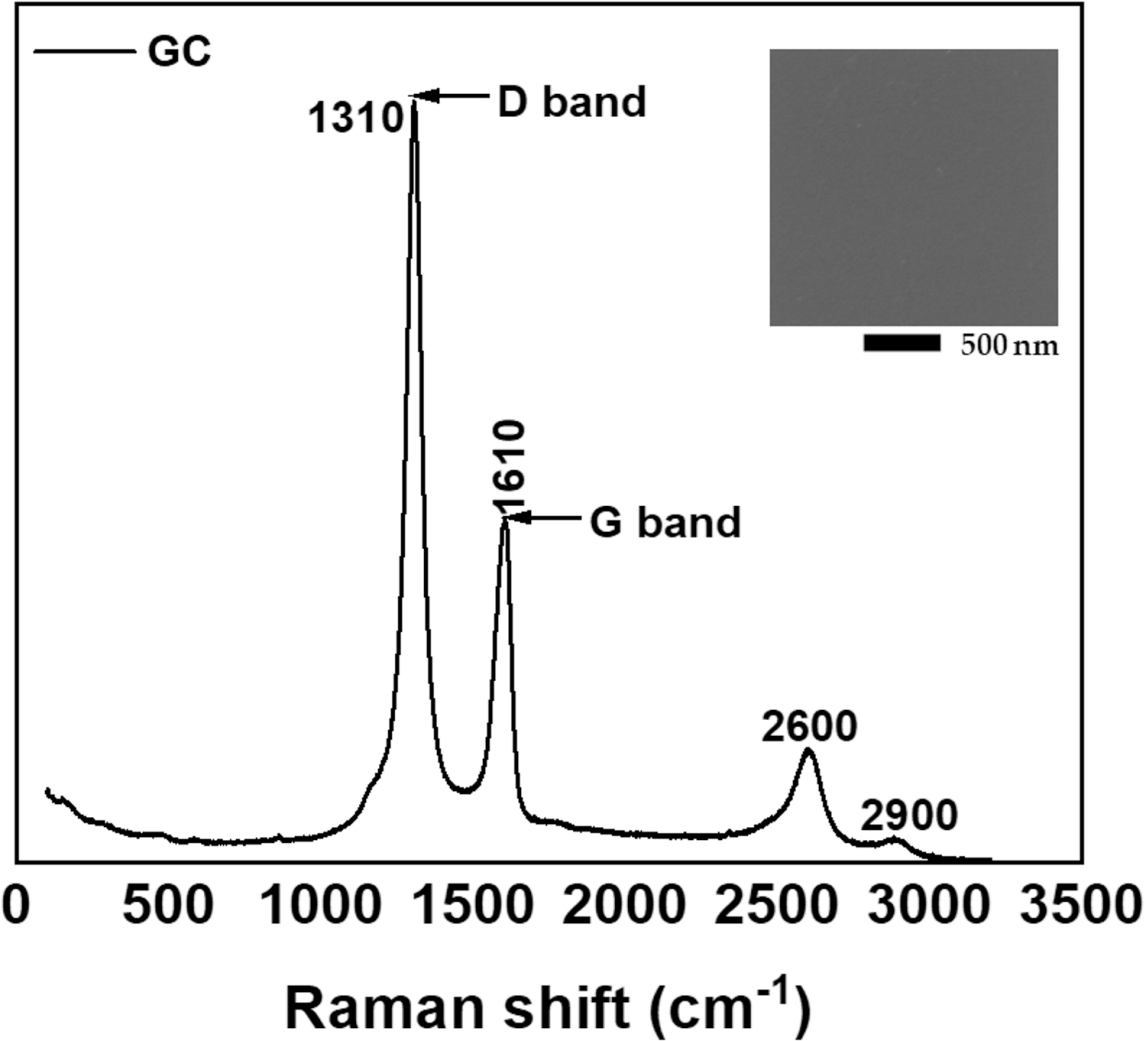
Raman spectrum of glassy carbon (GC) measured under a 785 nm excitation wavelength. The inset shows D band (1310 cm^–1^) and G band (1610 cm^–1^) peaks of graphitic carbon.

### Synthesis of PANI1 and PANI2

The synthesis of PANI exhibits a strong dependence on the reaction medium. The concentrated formic acid plays a pivotal role in PANI synthesis via facilitating electron transfer between the monomer (aniline) and the initiator (APS). The synthesis of PANI involves the reaction between the Lewis base aniline and the Bronsted–Lowry acid (formic acid). This initial step likely does not necessarily involve a simple neutralization reaction. Instead, formic acid acts as a proton transfer agent, facilitating the protonation of the aniline. The resulting anilinium cation then participates in the subsequent oxidative polymerization initiated by APS. This polymerization process can lead to the formation of two types of PANI backbones: one with oxidized nitrogen and another with reduced nitrogen. These variations are mainly attributed to the aniline-to-APS ratio. A higher APS concentration in the system promotes the growth of PANI2, which polymerizes faster than PANI1 (Figure 1). This is attributed to the higher content of APS, which promotes the formation of cation radicals with the following coupling of aniline monomers.

### SEM and TEM of PANI1 and PANI2

The SEM analysis of PANI1 and PANI2 films deposited on glassy carbon revealed distinct morphological characteristics (Figure 3). PANI1 exhibits a porous structure with nanofibril-like features (Figure 3(a),(b)), whereas PANI2 displays densely packed features (Figure 3(c),(d)). Oligomers with molecular weights corresponding to 6–10 aniline units were identified in both PANI samples. These oligomers self-assemble into supramolecular nano-objects.

**Figure 3:**
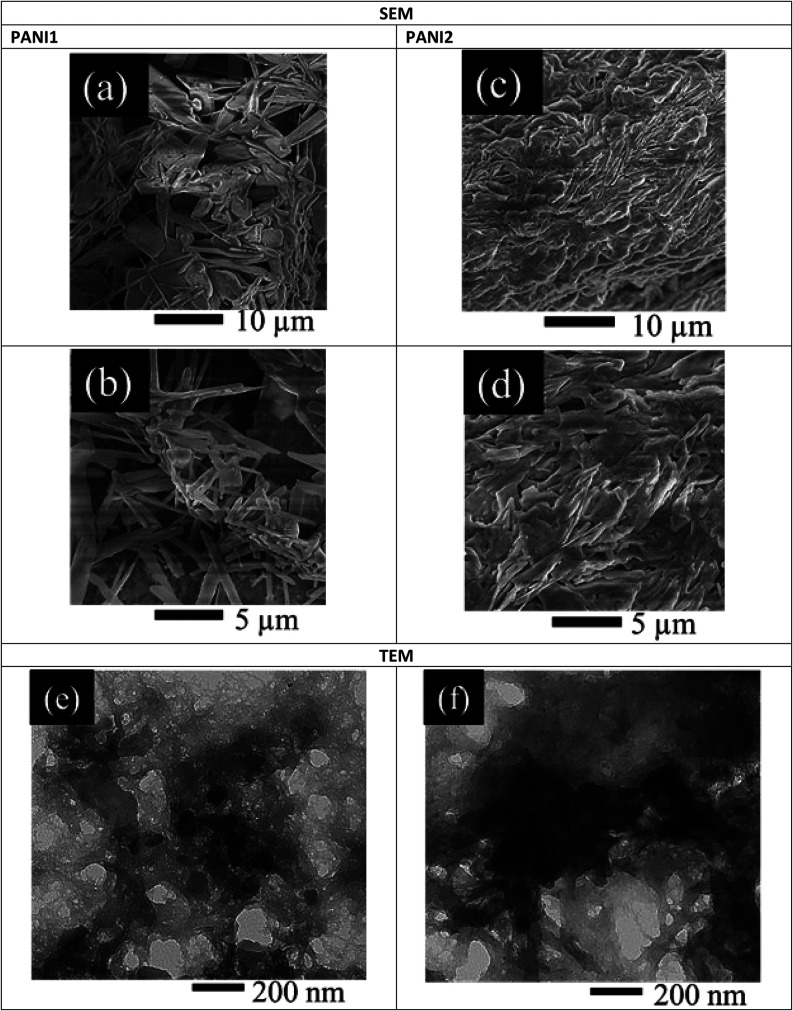
SEM images of PANI1 at (a) low and (b) high magnifications and PANI2 at (c) low and (d) high magnifications deposited on glassy carbon. TEM images of (e) PANI1 and (f) PANI2 at 200 nm.

Notably, PANI exhibited thinner nanofibrils compared with PANI2, a phenomenon attributed to the differences in polymerization kinetics. The slower polymerization rate of PANI1 facilitated a more controlled chain growth and subsequent assembly into well-defined nanostructures. Regardless of the similarity of the chemical structures and chain lengths of PANI1 and PANI2, the rapid polymerization of PANI2 resulted in less organized and more disordered nano-object morphologies. TEM analysis corroborated the SEM findings, also revealing distinct morphologies. Unlike PANI2, which had a morphology made up of larger, less organized aggregates, PANI1 displayed a more open, scaffold-like structure made up of thinner nanofibrils. These observations are consistent with the proposed reaction kinetics, as explained in the SEM, where a slower polymerization rate of PANI1 fosters the controlled diffusion of monomers into finer, well-dispersed, and nanofibrillar structures. In contrast, PANI2 seemed to undergo rapid polymerization, resulting in uncontrolled chain growth and entanglements, which hindered order and also promoted larger, irregular aggregates. While both PANI variants have similar chemical structures and chain lengths, the polymerization kinetics played a crucial role in defining the nanoscale morphology. To be more specific, the web-like architecture of PANI1 TEM micrographs displayed features with smoother contours and less aggregation, indicating a greater surface area and porosity than PANI2. However, PANI2 had a much lower surface area due to dense portions with larger surface area clusters, indicating more interconnected porosity.

### Raman, XPS, and TGA Measurements of PANI1 and PANI2

The chemical compositions and structures of PANI1 and PANI2 were investigated by Raman spectroscopy with an excitation line of 785 nm. Raman spectra with labeled most prominent peaks are shown in Figure 4(a),(b). The most notable peaks in the Raman spectra of PANI1 and PANI2 are observed at ∼1600 and 1500 cm^–1^, corresponding to the C–C stretching vibrations of semiquinoid rings and –C = N– stretching imine sites.^26,42^ The 1347–1360 cm^–1^ peaks indicate the presence of semiquinone cation radicals in delocalized polaronic structures and C–N+ stretching vibrations in highly localized polarons.^43^ The peak at around 1180 cm^–1^ is attributed to the in-plane C–H bending in the quinoid unit.^44^

**Figure 4:**
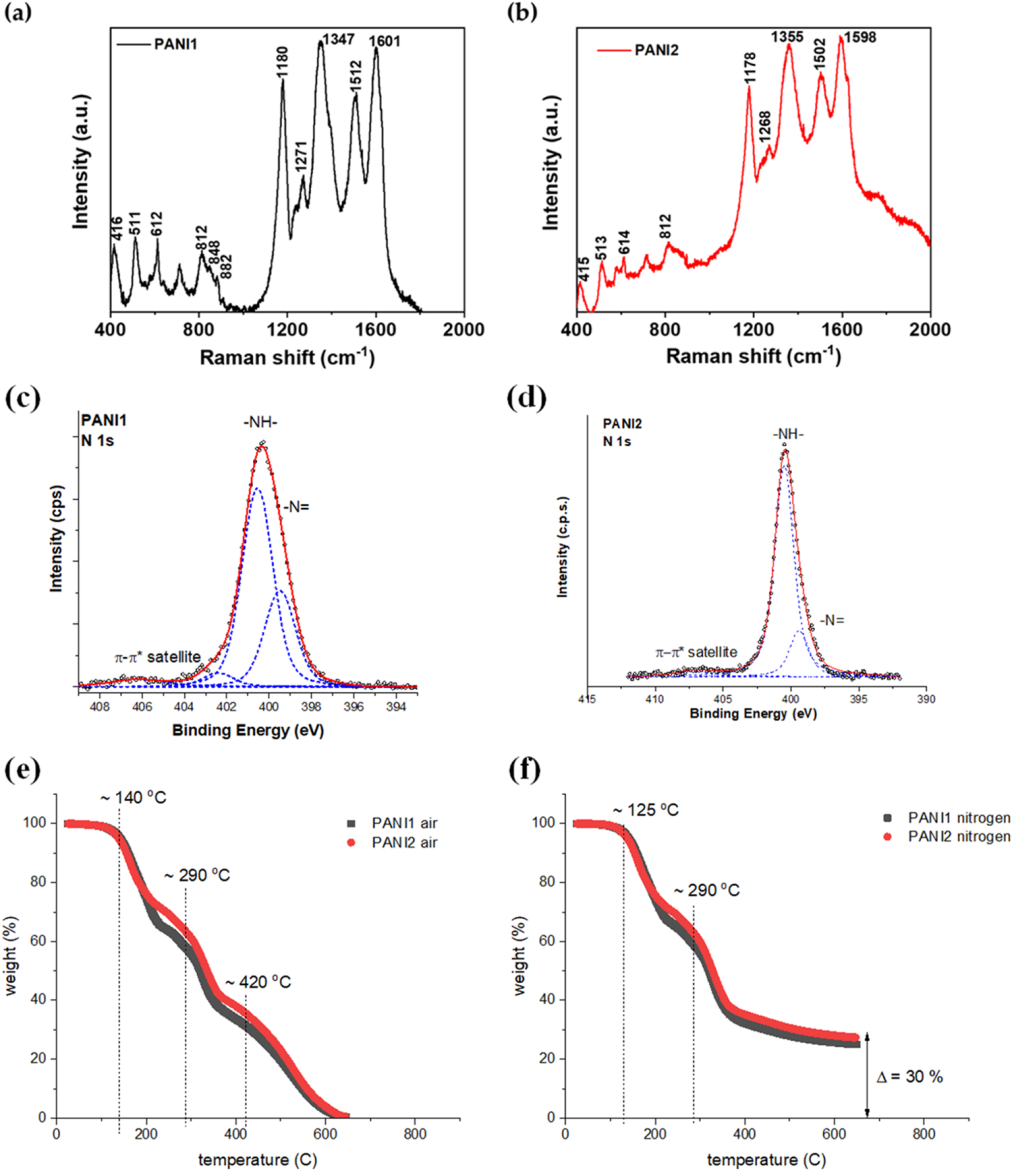
Raman spectra of (a) **PANI1** and (**b**) **PANI2** on glassy carbon measured by a 785 nm excitation laser. X-ray photoelectron spectroscopy of (c) PANI1 and (d) PANI2. TGA data for (e) PANI1 and PANI2 in air and (f) PANI1 and PANI2 in nitrogen.

From the Raman spectra, it can be confirmed that both polymers have comparable chemical structures and compositions. Additionally, Table 1 details the secondary peaks plotted in Figure 3(a),(b).

**Table 1:** Overview of Raman Bands of **PANI1** and **PANI2** and Their Assignment Adopted from refs 43–45

band position cm^–1^			band position cm^–1^		
PANI1	PANI2	assignment	PANI1	PANI2	assignment
416	415	out-of-plane ring deformations in the emeraldine base	1271	1268	C–N stretching in quinonoid structures
511	513	out-of-plane ring deformations in the emeraldine base	1347	1355	C–N+* stretching vibrations of the semiquinone cation radicals in delocalized polaronic structures
612	614	phenylene ring torsion and phenazine-like cross-linking	1396	1425	C–N+* ring-stretching vibrations, C–N+* stretching vibrations in highly localized polarons
712	713	C–C ring deformation vibration (out-of-plane) of polaronic form		1448	C–N stretching in highly localized polaronic structures, C = N stretching vibrations in quinonoid units
812	812	the benzene ring deformation in the emeraldine salt	1512	1502	N–H deformation in the semiquinonoid structures
851		C–C ring deformation vibration (out-of-plane) in the quinonoid ring	1533	1527	C = C stretching vibrations of the quinonoid ring
848	884	C–C ring deformation vibration (out-of-plane) in the polaronic form	1601	1598	C–C stretching vibrations of the semiquinonoid ring, C = C stretching vibrations in the quinonoid ring
			1624	1624	C–C stretching vibrations of the phenylene ring
1180	1178	C–H deformation in-plane vibrations of quinonoid and benzenoid rings			phenazine-like cross-linking
1236	1238	C–N stretching in benzenoid units			

The X-ray photoelectron spectroscopy (XPS) analysis was conducted to further characterize the chemical structures of PANI1 and PANI2; particularly, the spectrum of N 1s is the most informative for PANI structure (shown in Figures 4(c),(d)). The deconvoluted peaks can be assigned as follows: the signal at ∼399.1 eV is assigned to the imine group – N= and the signal at ∼400.6 eV is assigned to the secondary amine group −NH–. The peak at 402.4 eV corresponds to positively charged nitrogen atoms. The XPS analysis confirms that PANI1 and PANI2 have similar chemical structures.

The thermogravimetric studies of PANI1 and PANI2 were conducted in both air and nitrogen flow, as shown in Figure 4(e),(f). The TGA was performed on dried PANI1 and PANI2 powders. Both polymers exhibit similar weight changes during the heating process, particularly the removal of formic acid (bonding to the PANI1 and PANI2 by hydrogen bonds) at ∼125–140 °C, which depends on the flowing gas. The PANI2 polymer loses ∼3% more weight compared to PANI1, likely due to its morphology. The second wave of weight loss is attributed to the removal of formic acid from the inner parts of the polymers. The decomposition of PANI1 and PANI2 in air starts at ∼420 °C, which is a much higher value than the reported literature values.^46,47^ The TGA of PANI1 and PANI2 performed in nitrogen flow reveals only two weight loss events, with a residual mass of ∼30%, which corresponds to the carbonization materials.

### Electrochemical Applications

To investigate the impact of PANI synthesis on its supercapacitor properties, the GC electrode was employed as a current collector. The GC electrodes were cleaned before PANI deposition by polishing with 0.05 μm alumina and washed with deionized water. Twenty microliters of both PANI1 and PANI2 suspensions was drop cast on the GC electrode with the same surface area.

### Cyclic Voltammetry (CV) Measurements of PANI1 and PANI2 on GC

CV measurements for PANI1 and PANI2 deposited on GC were investigated in a three-electrode cell configuration with 5 M H_3_PO_4_ as the electrolyte, as shown in Figure 5(a). Figure 5(a) illustrates a comparative electrochemical behavior of PANI1 and PANI2 films at 10 and 30 mV/s scan rates. Additionally, Figure 5(b) shows the specific capacitance (F/g) of PANI1 and PANI2 on the GC electrode calculated from CV measurements by using eq 1.

**Figure 5:**
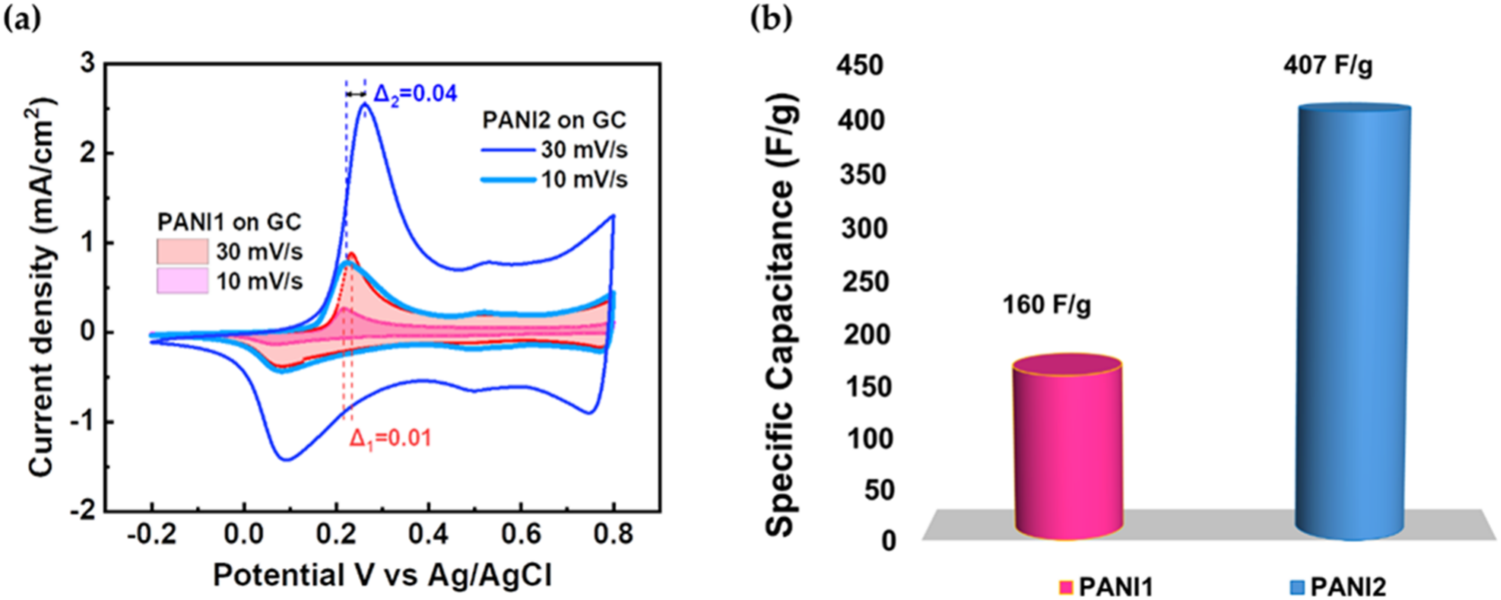
(a) Cyclic voltammetry curves of PANI1 and PANI2 on the GC electrode measured in 5 M H_3_PO_4_ at scan rates of 10 and 30 mV/s. (b) Specific capacitance of PANI1 and PANI2 on the GC electrode.

A quasi-reversible Faradic process is evident, characterized by both oxidation and reduction peaks. As the scan rate increases from 10 to 30 mV/s, the oxidation potential shifts from 0.22 to 0.23 V for PANI1 and from 0.22 to 0.26 V for PANI2. In contrast, the reduction peaks do not exhibit a scan rate dependence. For PANI2, the more significant shift in the oxidation potentials (0.04 V) seems to be attributed to the physical–chemical properties of the PANI/GC heterostructure, in our case related to PANI2’s densely packed structure and optimized charge transfer at the GC support. This unique combination makes the PANI2/GC electrode more suitable for electrochemical storage applications in comparison to the PANI1/GC electrode.

### Electrochemical Impedance Spectroscopy (EIS) of PANI1 and PANI2 on GC

EIS was employed to investigate the interaction between PANI and the GC support. It provides complementary information to that obtained from CV measurements.^48^ The results are presented as the Nyquist and Bode plots in Figure 6(a),(b) for PANI1/GC, and Figure 6(c),(d) for PANI2/GC, respectively. The PANI2/GC electrode revealed a substantially lower impedance and phase angle φ at low frequency compared to its PANI1 counterpart. The phase angle φ, ranging from 0 to −90°, deviates from ideal capacitive behavior, indicating nonideal capacitive characteristics or the presence of an ion-diffusion control process. This deviation is attributed to the electrode’s morphological features, such as roughness and porosity, which can influence charge storage mechanisms and ion transport processes. Notably, the porous morphology of PANI1 resulted in a phase angle φ value of 75° in contrast to 45° for PANI2. The total impedances of PANI1/GC (Figure 6(b)) and PANI2/GC (Figure 6(d)) are similar in the frequency range from 10 kHz to 50 Hz, which indicates that the charge transfer resistance between the GC and PANI is identical. However, the imaginary part of the impedance for PANI2/GC is 3.5 times smaller compared to that of PANI1/GC (see Figure 6(a),(c)). These findings suggest that the PANI2/GC electrode showed superior electrochemical properties for supercapacitor applications. A reduced impedance correlates with enhanced performance. This electrochemical behavior is attributed to the inherent properties of the GC electrode.

**Figure 6:**
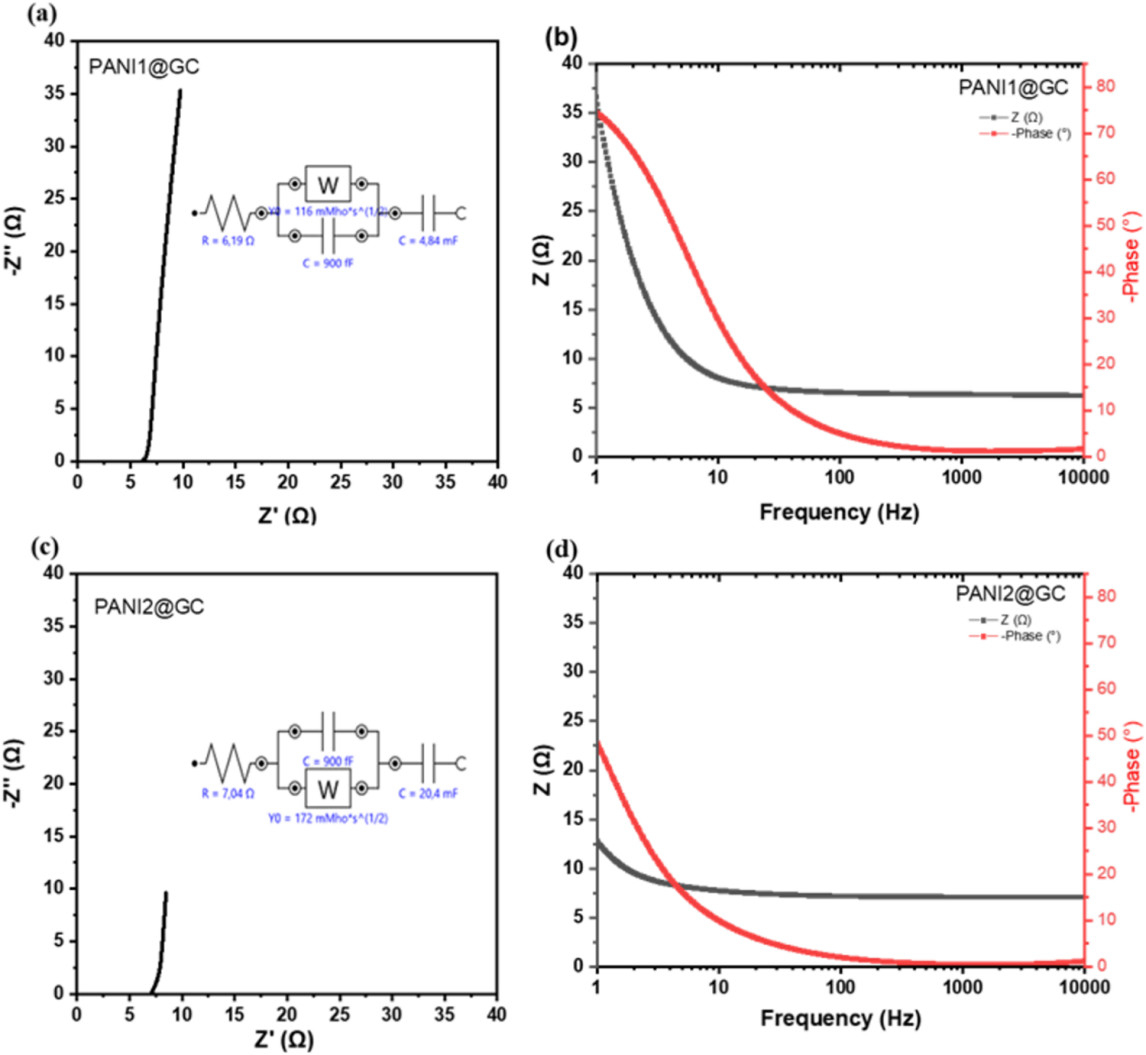
EIS data plotted in the form of the Nyquist plots for (a) PANI1/GC and (c) PANI2/GC and Bode plots for (b) PANI1/GC and (d) PANI2/GC.

The equivalent circuit model is proposed and presented in Figure 6(a),(c). The model comprises the solution resistance (identical for both polymers), a Warburg element (representing the diffusion of ions into the porous electrode in the intermediate frequency region), and two capacitances (double layer and Faradic one). The lower value for the Warburg impedance for PANI1 confirms that the diffusion of ions through the electrode/electrolyte interface is facile compared with the PANI2 interface. Moreover, the Faradic capacitance for PANI2 is 4 times that of PANI1, which corroborates *the* CV measurement results (Figure 5(b)).

### Specific Capacitance

The specific capacitance (calculated from CV using eq 1) is 160 F/g for PANI1 on GC and 407 F/g for PANI2 on GC, which is 2.5 times higher than that of PANI1 (Figure 5(b)). This difference is likely attributed to the surface morphology of the PANI1 and PANI2 electrodes and the densely packed morphology of PANI2. While the experimentally determined specific capacitance of PANI2 on GC reached 407 F/g, this value remains significantly lower than the theoretically predicted value (900 F/g). The theoretical value of specific capacitance was calculated using eq 2, where the number of electrons participating in the oxidation/reduction process was 1, with the working potential window of 1 V. From the EIS data (Figure 6(a),(c)), the limiting capacitance can be calculated using eq 3. The limiting capacitance values for PANI1/GC and PANI2/GC are 100 and 730 mF/cm^2^, respectively, calculated at the frequency of 1 Hz. The limiting capacitance for PANI2/GC is 7 times that of PANI1/GC, which does not correlate with CV measurements (where the specific capacitance of PANI2/GC is 2.5 times higher than that of PANI1/GC). The discrepancy between the limiting capacitance values obtained from EIS and CV measurements can be attributed to the chosen method and applied frequency. Nevertheless, both methods confirm that PANI2 exhibits higher specific and limiting capacitances compared to PANI1. Further investigations are envisioned to elucidate the underlying mechanisms of the synthesis of PANI and its interaction with the support material. Raman spectroscopy and crystallographic analyses of the PANI solution will provide valuable insights into the material’s vibrational properties and structure. Given the presence of crystalline traces observed for PANI2 in SEM, photoluminescence studies can be effective for understanding the optical properties and potential excitonic processes. Optimizing the synthesis process with varying mass loadings will enable a systematic evaluation of PANI’s influence on the support material electrodes. The use of electrodes with lower resistivity is recommended to minimize the unwanted effects of the support’s electrical resistance on the electrochemical measurements.

## Calculation

All electrochemical measurements were performed in a three-electrode cell configuration, and the calculations correspond to such a setup. The following equation calculates the specific capacitance (*C*_s_)­

1 $$C_{S}=\frac{\int i\cdot vdv}{2\mu \cdot m\cdot \Delta V}\left[F/g\right]$$

where *i* and v are current and potential in the CV test, respectively; μ represents the scan rate (V/s); the mass of the active materials is indicated as *m* in (g); and Δ*V* is the potential window in (V).

The following equation can calculate the theoretical capacitance

2 $$C=\frac{n\cdot F}{\left(M\cdot \Delta V\right)}$$

where *n* is the number of electrons, *F* is the Faraday constant (96 485 C/mol), Δ*V* is the potential window in (V), and M is the molecular mass of the monomer units (g/mol).

The limiting capacitance was calculated by using

3 $$C^{\prime \prime }\left(\omega \right)=Z^{\prime }\left(\omega \right)/(\omega \times |{Z\left(\omega \right)|}^{2}\left[F/{\mathrm{cm}}^{2}\right]$$

where *Z*′ is the real part of the impedance (Ohm cm^2^), ω is the angular frequency (ω = 2π*f*, *f* is the frequency Hz), and *Z*(ω) is the total impedance.

## Materials and Methods

### Materials

Analytical grade aniline, ammonium peroxydisulfate, APS (99% Lachner, Czech Republic), concentrated formic acid (98% Sigma-Aldrich, Czech Republic), *ortho*-phosphoric acid (85%, Merck, Czech Republic), and ethanol were used as received without further purification.

### Synthesis of Polyaniline

The PANI solution was synthesized according to the following procedure at an ambient temperature (20–25 °C). A 0.3 M aniline (0.2 mL) was dissolved in 1 mL of ethanol and about 5 mL of concentrated formic acid and mixed with 0.047 g of 0.03 M APS dissolved in 1 mL of deionized water (aniline-to-APS ratio of 10:1). The resulting solution was labeled PANI1. Under the same conditions, PANI2 was synthesized by doubling the amount of APS to 0.094 g (aniline/APS ratio of 5:1). Doubling the amount of APS accelerated the polymerization rate of PANI2 compared to PANI1.

### Characterization of the PANI Solution

The surface morphology of PANI films deposited on GC substrates was investigated by using a scanning electron microscope (SEM). Imaging was performed using a MAIA 3 SEM system (Tescan Ltd., Czech Republic) with an acceleration voltage of 10 kV in a top-view configuration. TEM observations were carried out at an accelerating voltage of 120 kV utilizing a bright field imaging mode on a Tecnai G2 Spirit Twin 12 instrument (FEI, Czech Republic). On a copper TEM grid (300 mesh) covered with a thin layer of electron-transparent carbon film, 3 μL of the solution was dropped. Filter paper was used to touch the grid’s bottom and remove any extra solution. Following 2 min of sedimentation, this quick solution removal was carried out to reduce oversaturation during drying. The sample was allowed to dry completely at room temperature prior to observation.

Using a Renishaw Via Raman microscope (Renishaw, U.K.) with a Peltier cooled CCD detector and 442 nm (Dual Wavelength HeCd laser, model IK5651R-G, Kimmon Koha) and 785 nm lasers for excitation (Diode laser, Renishaw HPNIR785 Laser Source), Raman spectra of PANI droplets were collected. A Leica objective 100× with NA 0.9 (50×/0.5 LWD) was used to gather scattered light in the confocal mode. For the utilized excitations, the spectrograph was equipped with holographic gratings measuring 2400 lines/mm and 1200 lines/mm, respectively. Each sample was subjected to a continuous wave laser with a power of 1.1 mW for 10 s at an average distinct site, allowing for the collection of average spectra per sample. The sample damage and changes in the material caused by light are avoided by using these parameters together. The average spectra were then determined, the baseline was removed using the asymmetric least-squares approach from the gathered spectra, and peak analysis was performed using Gaussian functions.

X-ray photoelectron spectroscopy (XPS) was measured on a K-alpha+ spectrometer (ThermoFisher Scientific; East Grinstead, U.K.) operating at a base pressure of 1 × 10^–6^ Pa. All samples were analyzed using a microfocused, monochromated Al Kalpha X-ray radiation (400 μm spot size) at an angle of incidence of 30° (measured from the surface) and an emission angle normal to the surface. The kinetic energy of the electrons was measured using a 180° hemispherical energy analyzer operated in the constant analyzer energy mode at 200 and 50 eV pass energy for the survey and high-resolution spectra, respectively. The data acquisition and processing were performed using Thermo Avantage software. All spectra were referenced to the C 1s peak of the hydrocarbons at 285.0 eV.

TGA data were collected at the Thermogravimetric analyzer Pyris 1 TGA (PerkinElmer). The measurements were performed in air or nitrogen with a heating speed of 5 °C/min.

A Metrohm AUTOLAB potentiostat/galvanostat driven by NOVA software was used to carry out the electrochemical measurements. A three-electrode cell configuration comprising a counter electrode (Pt), a working electrode (fabricated PANI/GC), and a reference electrode (Ag/AgCl, 3 M KCl) was utilized for the CV measurements at a scan rate of 10 and 30 mV/s. The potential ranged from −0.2 to 0.8 V vs Ag/AgCl.

The fitting of the EIS data was performed by using NOVA software.

## Conclusions

In summary, a PANI synthesis protocol via an acid-assisted polymerization technique has been successfully established. The concentration of APS significantly influenced the reaction kinetics, resulting in accelerated polymerization with an increased concentration of the initiator. SEM characterization revealed distinct morphological features: PANI1 displayed porous nanofibril-like structures, while PANI2 exhibited densely packed structures. The SEM images of the GC support confirmed a smooth and homogeneous material. The electrochemical data suggest that the oxidation potential’s position depends primarily on the morphology of the PANI films, the current collector, and the applied electrolyte. In contrast to PANI1, the enhanced interfacial interaction between the support and PANI2 can be attributed to the synergistic effects, including the sp^2^-carbon content of the GC electrode and the closely packed morphological structure of PANI2, resulting in a more effective charge transfer and a specific capacitance of 407 F/g.

## Data Availability

All data generated during this study are available at 10.5281/zenodo.13772941.
